# Procedural Outcome and 1-Year Follow-Up of Young Patients Undergoing Implantable Cardioverter–Defibrillator Implantation—Insights from the German DEVICE I+II Registry

**DOI:** 10.3390/jcm13133858

**Published:** 2024-06-30

**Authors:** Da-Un Chung, Matthias Hochadel, Jochen Senges, Thomas Kleemann, Lars Eckardt, Johannes Brachmann, Gerhard Steinbeck, Robert Larbig, Christian Butter, Thomas Uher, Stephan Willems, Samer Hakmi

**Affiliations:** 1 Department of Cardiology & Critical Care Medicine, Asklepios Klinik St. Georg, 20099 Hamburg, Germany; s.willems@asklepios.com; 2 Semmelweiß University Budapest, Asklepios Campus Hamburg, 20099 Hamburg, Germany; s.hakmi@asklepios.com; 3 Stiftung Institut für Herzinfarktforschung, 67063 Ludwigshafen am Rhein, Germanysenges@stiftung-ihf.de (J.S.); 4 Medizinische Klinik B, Ludwigshafen Hospital, 67063 Ludwigshafen am Rhein, Germany; kleemant@klilu.de; 5 Department of Cardiology II–Rhythmology, University Hospital Munster, 48149 Münster, Germany; lars.eckardt@ukmuenster.de; 6 Medical School/RegioMed GmbH, 96450 Coburg, Germany; johannes.brachmann@regiomed-kliniken.de; 7 Center for Cardiology, Starnberg Klinik, 82319 Starnberg, Germany; g.steinbeck@kardiozentrum-starnberg.de; 8 Department of Cardiology & Critical Care Medicine, St. Franziskus Hospital, Kliniken Mariahilf GmbH, 41063 Mönchengladbach, Germany; robert.larbig@mariahilf.de; 9 Department of Cardiology, University Hospital Heart Centre Brandenburg, Brandenburg Medical School (MHB), 16816 Bernau, Germany; christian.butter@mhb-fontane.de; 10 Department of Cardiology, General Hospital Celle, 29223 Celle, Germany; thomas.uher@akh-celle.de; 11 Department of Cardiac Surgery, Asklepios Klinik St. Georg, 20099 Hamburg, Germany

**Keywords:** implantable cardioverter–defibrillator, sudden cardiac death, young patients, device therapy, heart failure, device complications

## Abstract

**Background**: The number of young patients receiving ICDs or CRT-Ds has been increasing in recent decades and understanding the key characteristics of this special population is paramount to optimized patient care. **Methods**: The DEVICE I+II registry prospectively enrolled patients undergoing ICD/CRT-D implantation or revision from 50 German centers between 2007 and 2014 Data on patient characteristics, procedural outcome, adverse events, and mortality during the initial stay and 1-year follow-up were collected. All patients under the age of 45 years were identified and included in a comparative analysis with the remaining population. **Results**: A total number of 5313 patients were enrolled into the registry, of which 339 patients (6.4%) were under the age of 45 years. Mean age was 35.0 ± 8.2 vs. 67.5 ± 9.7 years, compared to older patients (≥45 years). Young patients were more likely to receive an ICD (90.9 vs. 69.9%, *p* < 0.001) than a CRT-D device (9.1 vs. 30.1%). Coronary artery disease was less common in younger patients (13.6 vs. 63.9%, *p* < 0.001), whereas hypertrophic cardiomyopathy (10.9 vs. 2.7%, *p* < 0.001) and primary cardiac electrical diseases (11.2 vs. 1.5%, *p* < 0.001) were encountered more often. Secondary preventive ICD was more common in younger patients (51.6 vs. 39.9%, *p* < 0.001). Among those patients, survival of sudden cardiac death (66.7 vs. 45.4%, *p* < 0.001) due to ventricular fibrillation (60.6 vs. 37.9%, *p* < 0.001) was the leading cause for admission. There were no detectable differences in postoperative complications requiring intervention (1.5 vs. 1.9%, *p* = 0.68) or in-hospital mortality (0.0 vs. 0.3%, *p* = 0.62). Median follow-up duration was 17.9 [13.4–22.9] vs. 16.9 [13.1–23.1] months (*p* = 0.13). In younger patients, device-associated complications requiring revision were more common (14.1 vs. 8.3%, *p* < 0.001) and all-cause 1-year-mortality after implantation was lower (2.9 vs. 7.3%, *p* = 0.003; HR 0.39, 95%CI: 0.2–0.75) than in older patients. **Conclusions**: Young patients < 45 years of age received defibrillator therapy more often for secondary prevention. Rates for periprocedural complications and in-hospital mortality were very low and without differences between groups. Young patients have lower mortality during follow-up but experienced a higher rate of postoperative complications requiring revision, potentially due to a more active lifestyle.

## 1. Introduction

Over recent decades, implantable cardioverter–defibrillator (ICD) and cardiac resynchronization therapy defibrillator (CRT-D) devices have evolved into irreplaceable mainstays in heart failure therapy and sudden cardiac death (SCD) prevention and are well-established in modern cardiovascular care [1,2,3]. Over recent decades, the number of ICD and CRT-D implantations have continuously increased, with a significant proportion of young patients receiving these devices [4,5]. Young patients present with relevant differences in device indications and comorbidities and have a longer life expectancy compared to the typically older cardiovascular patient; thus, they represent a unique subgroup with specific challenges for lifetime and complication management [6,7,8]. Current guideline recommendations for primary and secondary prevention of SCD with ICDs/CRT-Ds are disease-specific and often do not sufficiently reflect patient-age-specific differences [1,2,9]. Recognizing and incorporating these differences into pre- and postprocedural patient care and management may increase patient safety, positive outcomes, and satisfaction [10,11,12]. In recent years, novel devices, such as the subcutaneous ICD (S-ICD), and various advancements in device programming and pharmacological treatment of heart failure have emerged. These advancements have provided young patients with new therapeutic options that better reflect age-appropriate needs in therapy and may be able to reduce the concomitant complications of device therapy or the need for device-based SCD-prevention itself [13,14,15]. The aim of this study was to analyze patient characteristics and procedural outcomes, as well as clinical outcomes after 1-year follow-up among young patients undergoing ICD/CRT-D implantation in Germany, in order to identify and define key differences for optimized patient care.

## 2. Material and Methods

### 2.1. Patient Recruitment and Data Collection

The German DEVICE I + II registry prospectively enrolled all patients undergoing ICD or CRT-D device implantation or revision from 50 participating centers under the aegis of the “Stiftung Institut für Herzinfarktforschung” (IHF, Ludwigshafen, Germany) between March 2007 and February 2014. The data was collected in a computerized database via an internet-based electronic case report form (eCRF) and analyzed retrospectively based on individual research inquiries and subgroups of interest of the participating centers. The database collected data on patient demographics, baseline characteristics, device indication, and periprocedural events. All patients gave written consent prior to enrollment and the registry was approved by the ethics committee of the state medical board of Rhineland-Palatinate (Landesärztekammer Rheinland-Pfalz, Mainz, Germany) with the processing-ID: 837.023.07 (5558) on 7 March 2007. The study complies with all regulations and statutes of the Declaration of Helsinki. This study focused on the analysis of patient characteristics, procedural outcomes, and outcomes during follow-up among patients younger than 45 years at the time of ICD/CRT-D implantation.

### 2.2. Follow-Up

The study included a prospective follow-up by telephone call at least 12 months after device implantation or revision, which was carried out by dedicated personnel of the IHF. The interview comprised a standardized set of questions regarding arrhythmic or cardiac events (e.g., syncope, myocardial infarction), hospitalizations, clinical symptoms, non-cardiac events, device-associated complications, changes in medication, and quality of life. In cases where the patient was unattainable by telephone, data was obtained from the patient’s general physician, cardiologist, or civil registration office. Additionally, data was collected from clinical records of follow-up visits and device interrogations at the participating centers, as far as these were available.

### 2.3. Statistical Analysis

Continuous variables are expressed as mean ± standard deviation (SD) for normal distributions and median with interquartile range (25th and 75th percentiles) for non-gaussian distribution. Categorical variables are shown as absolute counts and percentages. Differences between groups were compared using Mann–Whitney-U test for continuous variables and χ^2^-test or Fisher’s exact test in cases of small event counts for categorical variables. The follow-up duration was defined as the time span from index discharge to the date of the follow-up contact, i.e., when information on the patient’s status was obtained. One-year survival was analyzed by the Kaplan–Meier method and a log-rank test. A two-tailed *p*-value < 0.05 was considered statistically significant. All statistical analyses were performed by the biometrics department of the IHF with the SAS statistics software package version 9.4 (Cary, NC, USA).

## 3. Results

### 3.1. Patient Characteristics

**Figure 1:**
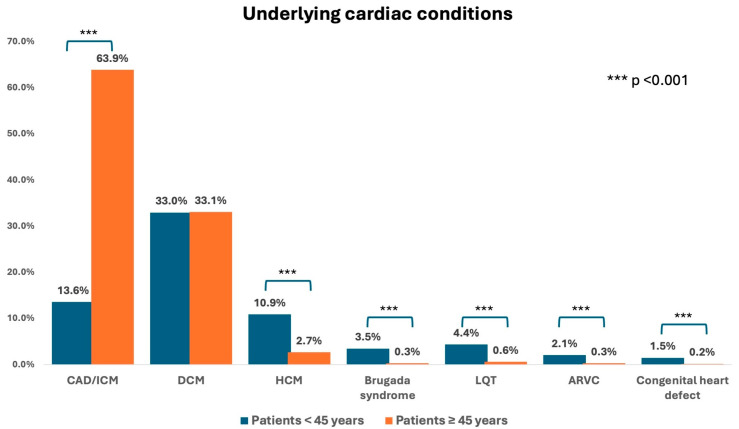
Bar diagram displaying the prevalence of underlying cardiac conditions between groups (blue: patients < 45 years; orange: patients ≥ 45 years). ARVC: arrhythmogenic right ventricular cardiomyopathy, CAD: coronary artery disease, DCM: dilatative cardiomyopathy, HCM: hypertrophic cardiomyopathy, ICM: ischemic cardiomyopathy, LQT: long QT syndrome. All *p*-values < 0.05 were considered statistically significant.

**Figure 2:**
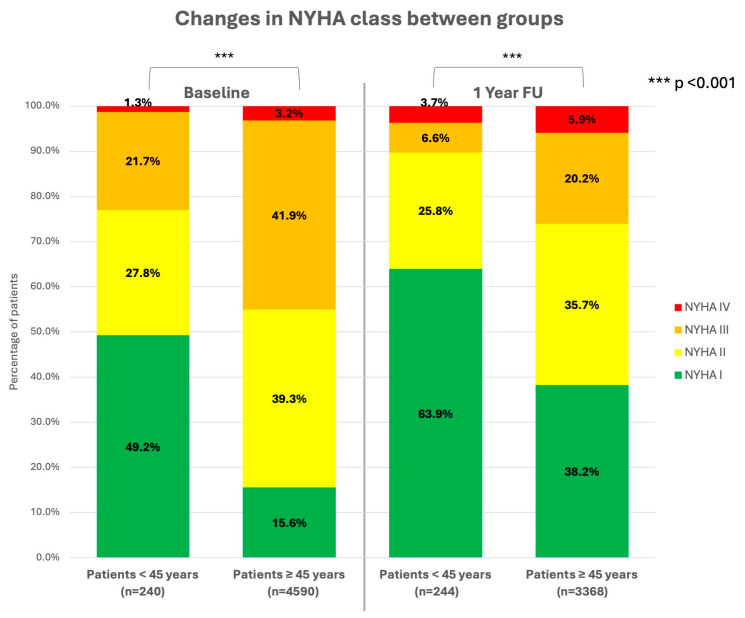
Bar diagrams displaying the distribution of NYHA classes in percent (green: class I; yellow: class II; orange: class III; red: class IV) at baseline and after 1 year follow-up between groups. A *p*-value < 0.05 was considered statistically significant.

The DEVICE I + II registry enrolled a total number of 5313 patients, of which 339 patients (6.4%) were younger than 45 years. The mean age was 35.0 ± 8.2 years, compared to 67.5 ± 9.7 years in patients 45 years or older. The prevalence of female patients was higher in young patients with 28.9 vs. 18.4% (*p* < 0.001). The median body mass index (BMI) was higher in the older patient group with 25.0 [21.9–28.5] vs. 27.1 [24.5–30.2] kg/m^2^ (*p* = 0.001). Median left ventricular ejection fractions (LVEF) between groups were 40 [25–60] vs. 30 [25–35] % (*p* < 0.001). Underlying cardiac condition was more prevalent in older patients (70.8 vs. 93.8%; *p* < 0.001), with coronary artery disease (CAD)/ischemic cardiomyopathy (ICM) being most common (13.6 vs. 63.9%; *p* < 0.001); of these, 8.3 vs. 36.6% (*p* < 0.001) had a history of prior myocardial infarction (MI) at presentation. History of myocardial revascularization (6.7 vs. 46.5%; *p* < 0.001) with either percutaneous coronary intervention (PCI) or coronary artery bypass graft (CABG) was also more common in the older patient group. Congestive heart failure (CHF) was more common in older patients (27.1 vs. 51.6%; *p* < 0.001). There was no difference in the prevalence of dilatative cardiomyopathy (DCM—33.0 vs. 33.1%; *p* = 0.98), but higher prevalence of hypertrophic cardiomyopathy (HCM—10.9 vs. 2.7%; *p* < 0.001) and congenital structural heart defects (1.5 vs. 0.2%; *p* < 0.001), as well as primary electrical diseases of the heart (11.2 vs. 1.5%; *p* < 0.001), in the younger patient group (see Figure 1). Most common NYHA class at presentation for young patients was class I (49.2 vs. 15.6%; *p* < 0.001), whereas older patients were most common in class III (21.7 vs. 41.9%; *p* < 0.001—see Figure 2). Non-cardiac comorbidities, such as history of cerebrovascular accident (CVA—2.1 vs. 4.2%; *p* = 0.057), peripheral artery disease (PAD—0.6 vs. 3.3%; *p* = 0.006), diabetes mellitus (3.5 vs. 28.7%; *p* < 0.001), chronic obstructive pulmonary disease (COPD—0.6 vs. 3.9%; *p* = 0.002), and chronic kidney disease (CKD—5.0 vs. 18.1%; *p* < 0.001) were more common in the older patient group. Younger patients most commonly presented with sinus rhythm (93.2 vs. 76.9%; *p* < 0.001). Atrial fibrillation was more prevalent in the older patient group with 3.5 vs. 19.6% (*p* < 0.001). Atrioventricular (AV) conduction disturbances (7.7 vs. 18.0%; *p* < 0.001) and left-bundle branch blockages (LBBB—11.8 vs. 33.5%, *p* < 0.001) were both documented in the older patient group more often. Median QRS-duration was 100 [90–120] vs. 120 [100–150] ms (*p* < 0.001) between groups. For more details, see Table 1 and Figure 1.

### 3.2. Device Indication and Characteristics

Regarding device indication, the ratio of primary to secondary prevention was 48.4/51.6% for young patients and 60.1/39.9% for older patients (*p* < 0.001), respectively. In cases of secondary prevention, the most commonly documented arrhythmia for young patients was ventricular fibrillation (VF) with 60.6%, whereas older patients suffered most from ventricular tachycardia with 46.8% of cases. Therefore, in those cases, the leading cause for initial admission was status post cardiopulmonary resuscitation (CPR) in 66.7% of young patients vs. 45.4% in older patients (*p* < 0.001). There was no difference in syncope (20.1 vs. 24.7%, *p* = 0.20), but presyncope was more common in older patients (5.0 vs. 15.7%, *p* < 0.001). Positive family history of sudden cardiac death was present in 15.2 vs. 3.9% of cases (*p* < 0.001) between groups (see Table 2). The most implanted device in both groups was the single-chamber (VVI) ICD (75.2 vs. 49.1%; *p* < 0.001). In patients aged < 45 years, it was followed by the dual-chamber (DDD) ICD (15.6 vs. 20.8%; *p* = 0.022), whereas in patients aged ≥ 45 years, the CRT-D (9.1 vs. 30.1%; *p* < 0.001) was the second most common device. Further data on device manufacturers can be seen in Table 3.

### 3.3. Procedural Results

In both groups, the majority of patients underwent de novo implantation of their respective devices with 89.6 vs. 85.7% (*p* = 0.046). Device revision was more common in patients aged ≥45 years (10.4 vs. 14.3%; *p* = 0.046). Most patients in both groups were treated as in-patients, but significantly more often in younger patients (97.9 vs. 89.9%; *p* < 0.001). The ratios of urgent vs. elective procedures between groups were 14.4/85.6% vs. 8.7/91.3% (*p* = 0.053). The median procedural duration was 46 [37–81] vs. 62 [41–105] minutes (*p* < 0.001). Defibrillation testing (DFT) was performed more often in younger patients (85.6 vs. 73.5%; *p* < 0.001), with most tests recorded as successful (99.6 vs. 99.4%; *p* = 0.61). Complications requiring intervention (1.5 vs. 1.9%; *p* = 0.68) did not differ between groups (see Table 3). Pneumothorax was most common (0.9 vs. 0.4%, *p* = 0.21) in the younger group, whereas pocket hematoma (0.3 vs. 1.3%, *p* = 0.19) was the most common complication requiring intervention for the older patients. Requirement for device revision before discharge (1.8 vs. 2.2%; *p* = 0.82) was also indifferent between groups (see Table 3). Reasons for revision for the younger group were RA- and RV-lead dislodgment in one case, respectively, and two cases of wound revision. Older patients most commonly experienced loss of sensing/capture or inacceptable increase in stimulation thresholds with 24.4% of cases and necessity for wound revision in 22.0%. Revision of the pulse generator, RA-, RV-, and coronary sinus leads (CS) were performed in 4.9%, 28.0%, 42.7%, and 15.9%, respectively.

### 3.4. Status at Discharge

The median duration of stay was 3 [2–5] vs. 3 [2–5] days (*p* = 0.28) between groups. There was no difference in all-cause in-hospital mortality (0.0 vs. 0.3%; *p* = 0.30). The prevalence of heart failure medication at discharge was lower for younger patients with 55.5 vs. 88.2% (*p* < 0.001) for angiotensin-converting enzyme inhibitors (ACEi)/angiotensin receptor-1-inhibitors (AT1i), 75.8 vs. 90.8% (*p* < 0.001) for betablockers, 23.0 vs. 40.5% (*p* < 0.001) for aldosterone–antagonists, and 33.9 vs. 74.0% (*p* < 0.001) for diuretics. Antiarrhythmic drugs were prescribed in 10.6 vs. 20.4% (*p* < 0.001) at discharge, with class III agents (5.3 vs. 14.2%, *p* < 0.001) being most common (see Table 3 and Figure S1).

### 3.5. Follow-Up

**Figure 3:**
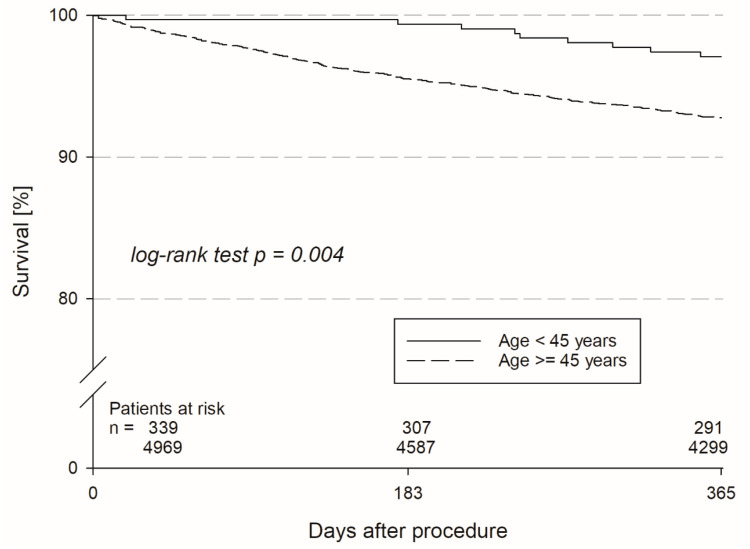
Kaplan–Meier estimates for survival during 1-year follow-up among patients aged < 45 years (dashed line) and patients age ≥ 45 years (continuous line). For patients aged < 45 years, the hazard ratio (HR) for death from any cause was 0.39 (95% CI: 0.20–0.75), compared to patients aged ≥ 45 years (log-rank test *p* = 0.004).

**Figure 4:**
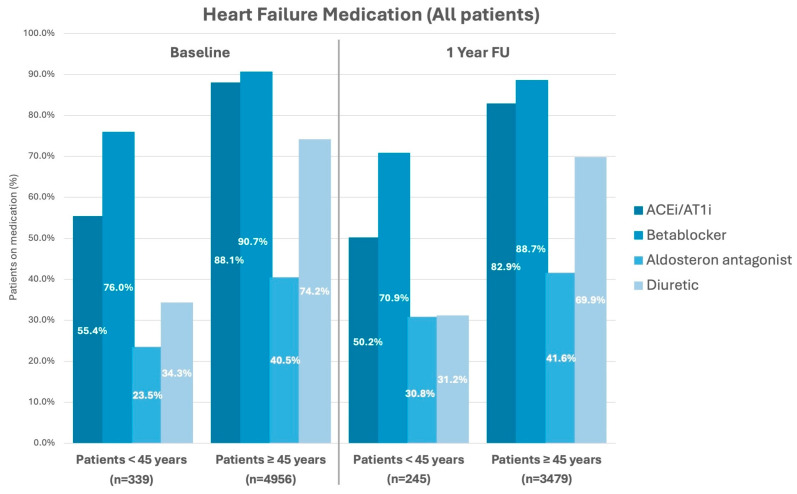
Bar diagrams displaying the frequency of different heart failure medications in percent between groups at baseline and after 1 year follow-up. ACEi: Angiotensin-converting enzyme inhibitor; AT1i: angiotensin receptor 1 inhibitor. A *p*-value < 0.05 was considered statistically significant.

Acquisition of follow-up (FU) data were achieved in 93.5% in the young patient group vs. 97.0% (*p* < 0.001) in the older patient group after a median duration of FU of 17.9 [13.4–22.9] vs. 16.9 [13.1–23.1] months (*p* = 0.13) between groups. During FU n = 11 (3.2%) patients in the young patient group had died, in contrast to n = 689 (13.9%) patients in the older group. That translated into a 1-year mortality of 2.9 vs. 7.2% (HR 0.39, 95%CI: 0.2–0.75; log rank *p* = 0.004) between groups, respectively (see Figure 3/Table 4). The cumulative incidence of death, MI, or stroke (MACCE—major adverse cardiovascular or cerebrovascular event) was 3.5 vs. 8.3% (*p* = 0.002). The causes of death were classified as “cardiovascular” in 36.4 vs. 26.8%, “non-cardiovascular” in 9.1 vs. 19.0%, and “unknown” in 54.5 vs. 54.2% (*p* = 0.98). There were no differences in non-fatal adverse events, such as MI (0.8 vs. 0.8%; *p* = 0.98), CVA (0.0 vs. 1.1%; *p* = 0.1), myocardial revascularization (1.0 vs. 2.7%; *p* = 0.15), or pulmonary embolism (PE—1.4 vs. 0.3%; *p* = 0.17). There were no differences in arrhythmia-associated complications, such as CPR (1.1 vs. 0.5%; *p* = 0.20), ICD shock (19.2 vs. 17.0%; *p* = 0.37), syncope (6.2 vs. 5.1%; *p* = 0.49), or VT storm/incessant VT (2.4 vs. 2.0%; *p* = 0.72) between groups. Heart failure symptoms stratified in NYHA classes decreased in both groups with an increased proportion of patients with none or mild symptoms (NYHA class I) of 63.9% in the younger group and 38.2% in the older group at FU (Figure 2). The proportion of patients with severe heart failure symptoms (NYHA III+IV) decreased accordingly from 22.9 to 10.2% in young patients and 45.1 to 26.1% in older patients. Rehospitalization occurred in 43.4 vs. 41.1% (*p* = 0.54) of cases with 22.6 vs. 13.9% (*p* < 0.001) being device-related rehospitalizations. Device revisions during FU was much more common in younger patients than older patients (overall: 13.7 vs. 8.1%; *p* = 0.002). Older patients suffered from more non-cardiovascular rehospitalizations (10.1 vs. 16.0%, *p* = 0.013) than young patients (Table 4). Median duration of all rehospitalizations were 7 [4–14] days vs. 10 [5–20] days (*p* = 0.023). At FU, heart failure medication was still present in 50.6 vs. 82.9% (*p* < 0.001) for ACEi/AT1i, 71.0 vs. 88.7% (*p* < 0.001) for betablockers, 30.6 vs. 41.6% (*p* < 0.001) for aldosterone–antagonists, and 31.0 vs. 69.8% (*p* < 0.001) for diuretics (see also Figure 4). After 1 year 51.7% of young patients had rejoined the work force again, whereas 16.1% were retired and 34.2% had “unknown” or “other” reported as employment status. Only 10.6% of older patients were still employed after 1 year and 73.2% had retired. Patient interviews at FU revealed, that the majority of patients in both groups deemed the therapy successful (92.1 vs. 91.1%, *p* = 0.70) and trusted in the protective capabilities of their defibrillator against SCD (95.9 vs. 93.8%, *p* = 0.84). However, younger patients were significantly more concerned about ICD shocks than older patients (48.6 vs. 20.5%, *p* < 0.001).

### 3.6. Subgroup Analysis of Patients with Congestive Heart Failure

**Figure 5:**
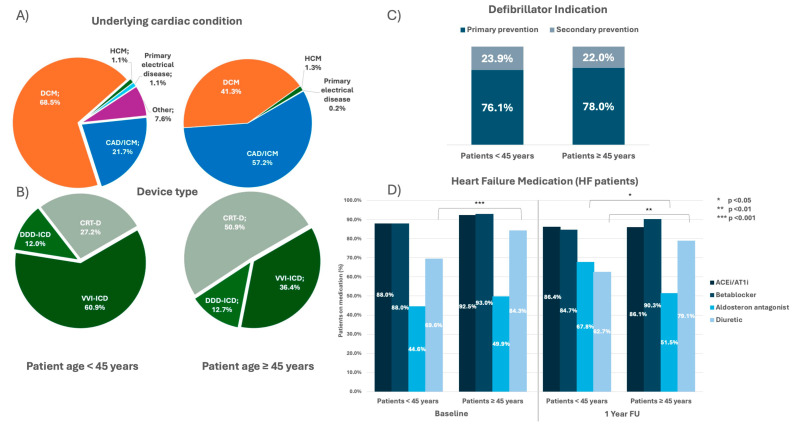
Patient and device characteristics of patients with history of heart failure (HF) stratified by age (patient age < 45 years, n = 92; patient age ≥ 45 years, n = 2569). (**A**) Pie diagram depicting proportion of underlying cardiac conditions between groups in %. (**B**) Pie diagram depicting proportion of device types between groups in %. (**C**) Bar diagram depicting the distribution of primary vs. secondary defibrillator indication between groups in %. (**D**) Bar diagrams displaying the frequency of different heart failure medications in % between groups at baseline and after 1 year follow-up. ACEi: angiotensin-converting enzyme inhibitor; AT1i: angiotensin receptor 1 inhibitor; CAD: coronary artery disease; CRT-D: cardiac resynchronization device with defibrillator; DCM: dilatative cardiomyopathy; DDD: dual-chamber; HCM: hypertrophic cardiomyopathy; ICM: ischemic cardiomyopathy; VVI: single-chamber. A *p*-value < 0.05 was considered statistically significant.

In order to gain a better understanding of heart failure patients in our cohort, we conducted a subgroup analysis of patients with history of CHF. We identified n = 92 patients in our younger cohort with a median age of 40 [34–42] years, who mostly presented with DCM (68.5%; n = 63), followed by CAD/ICM (21.7%; n = 20), as underlying cardiac conditions. Primary electrical diseases were only encountered in 1.1% (n = 1) of cases (see Figure 5). Compared to older defibrillator patients, median LVEF was indifferent between groups with 25.0 [20–32] % vs. 25.0 [20–30] % (*p* = 0.85). Defibrillator indication was primary preventive in 76.1 vs. 78.0% (*p* = 0.67) and secondary preventive 23.9 vs. 22.0% (*p* = 0.67), respectively. Most young patients received VVI-ICD (60.9 vs. 36.4%; *p* < 0.001), whereas CRT-D was the most common device for older patients (27.2 vs. 50.9%; *p* < 0.001). The prevalence of LBBB was 30.4 vs. 49.6% with a median QRS width of 102 [100–132] ms vs. 140 [110–160] ms (*p* < 0.001). Proportions of patients with a QRS width ≥150 ms were 19.8% and 43.1% (*p* < 0.001), respectively. In-hospital mortality (0.0 vs. 0.3%; *p* = 1.00) and periprocedural complications requiring intervention (0.0 vs. 2.0%; *p* = 0.42) were indifferent between groups. The prevalence of heart failure medication at discharge was high in both groups and remained so during FU (see Figure 5). One-year mortality during FU was indifferent between groups (6.3 vs. 8.2%; HR: 0.74, 95% CI: 0.31–1.81; *p* = 0.51). There were no differences in rehospitalizations (45.0 vs. 43.6%; *p* = 0.83) or revision surgeries (9.5 vs. 9.0%; *p* = 0.88).

## 4. Discussion

This study could demonstrate that young patients represent a small but still significant number of patients who undergo ICD/CRT-D implantation in Germany. The main findings are as follows: (A) this patient population frequently present with HCM or primary electrical diseases of the heart as underlying conditions; (B) this patient population more often receive defibrillator therapy for secondary prevention; (C) this patient population receive CRT-D less often, even in heart failure; (D) this patient population has lower rates for MACCE and mortality after 1-year follow-up; (E) this patient population more often require device revision compared to older patients.

### 4.1. Defibrillator Indication

Our results show that young patients receive defibrillator implantation mostly due to an unacceptably high risk of SCD or as SCD survivors, with few–no concomitant comorbidities. In contrast, older patients receive ICD or CRT-D therapy mainly due to ICM, its risk factors and sequelae. The significantly increased proportion of young patients receiving defibrillator therapy for secondary prevention (51.6 vs. 39.9%) due to status post-CPR on initial admission (66.7 vs. 45.4%), the high prevalence of patients without underlying cardiac disease (29.2 vs. 6.2%) and positive family history for SCD (15.2 vs. 3.9%), compared to older patients in our study, support this notion. The baseline characteristics (Table 1) of our young study population fit well with registry data of patients with inherited arrhythmia syndromes [6,10], but are not well reflected in the landmark trials that formed current guidelines on SCD prevention and heart failure [1,2].

Almost half of our young patient cohort (48.4%) received defibrillator implantation for primary prevention, which is more than usual for patients in that age group in the literature (range 38–43%) [6,10]. Most landmark trials for the primary prevention of SCD have focused on older patients with highly reduced LVEF, such as MADIT II for ICM, DEFINITE for non-ischemic cardiomyopathy (NICM), and SCD-HeFT for both (median age: 58–66 years), which were all able to demonstrate a significant reduction in mortality with ICD compared to medical therapy at that time [16,17,18]. Whereas the indication for primary prevention in ICM with reduced LVEF has remained undisputed, the DANISH trial in 2016 questioned the benefit of ICD in NICM solely based on LVEF and demonstrated a survival benefit of primary preventive ICD only in a subgroup of patients aged < 59 years [19]. The advent of modern heart failure medication, such as angiotensin receptors/neprilysin inhibitors (ARNIs) [20] and sodium glucose transporter 2 (SGLT2) inhibitors [21] and their ability to reduce SCD-risk independently from an ICD has questioned the indication of primary preventive ICD even further, especially for patients with NICM [22]. Shen et al. were able to demonstrate a 44% decline of SCD risk across 12 heart failure trials spanning a period from 1991 to 2014 [15], even before the introduction of SGLT2 inhibitors, which underlines the constant need to further review and redefine our current practice.

Cardiac resynchronization therapy (CRT) with and without defibrillator function in addition to heart failure medication has been repeatedly shown to reduce mortality in patients with heart failure [23,24]. But there is still ongoing debate as to whether the survival benefit is independent of defibrillation therapy or not [25]. The prevalence of CRT-D was low, with 9.1% in younger patients, and stayed low, with 27.2% in our subgroup analysis of CHF patients only (see Section 3.6); this might be attributed to the lower frequency of relevant LBBB in younger patients (Table 1). The DANISH trial could demonstrate no added survival benefit of CRT-D over a CRT-Pacemaker (CRT-P) for NICM patients and the observational part of the RESET-CRT study was able to show the same in an age- and comorbidity-adjusted cohort of ICM and NICM patients [26]. 

With 33.1% in the overall cohort and 68.5% in the CHF cohort, DCM was the most common underlying cardiac condition in our younger patient group and most, if not all patients received ICD/CRT-D before these crucial heart failure therapeutics were fully implemented in clinical practice. Thus, the question remains, how many of these patients would have been spared from an ICD after 3 months of contemporary heart failure medication and if those with CRT-D would have been better served with a CRT-P, instead. 

The decision for primary prevention in HCM and inherited arrhythmia syndromes is currently based on clinical, genetic, and electrocardiographic risk markers; in the case of HCM, long QT syndrome and arrhythmogenic right-ventricular cardiomyopathy (ARVC) have been incorporated into a specific risk scores in order to aid decision making [1,27,28,29].

Slightly more than half (51.6%) of the young patient cohort in our study was implanted for secondary prevention. A meta-analysis of three early secondary prevention trials, mostly in ICM patients, could demonstrate a mortality reduction of 28% compared to pharmacotherapy alone, which was mainly driven by the prevention of SCD in the ICD group [30]. But current evidence on secondary prevention in NICM, HCM, and primary cardiac electrical diseases suggests that an ICD after survival of SCD in the absence of reversible causes seems to be without an alternative, and remains a class I recommendation in the respective guidelines, regardless of patient age and underlying etiology [1,3].

### 4.2. Device Type and Procedure

The distributions of VVI- or DDD-ICD and CRT-D devices in our younger patient cohort were 75.2%, 15.6%, and 9.1%, respectively, which are comparable to contemporary registry data on young ICD recipients in northern Europe [6]. Defibrillation testing (DFT) was performed very frequently in both groups, but significantly more often in the younger cohort (85.6 vs. 73.5%); this may be explained by the higher proportions of patients receiving their ICD as secondary prevention and higher prevalence of with HCM or primary electrical diseases. The current guidelines [31] allow for the omission of DFT in the setting of left-sided pectoral implantation and anatomically well-placed defibrillator leads, but two trials (SIMPLE and NORDIC-ICD) [32,33] that were instrumental in shaping these recommendations had not been published yet, which may have led to more frequent testing in both groups at that time. Moreover, cardiac conditions other than ICM or NICM were not well represented in these trials, so it is fair to assume that a significant proportion of our patients in the young cohort still would have undergone DFT, if implanted today.

Complications requiring intervention were 1.5% in the young patient group and 2.3% in the older group, which was lower than in larger ICD registries, with a reported frequency of 3.2% [34]. The increased rate of pocket hematomas in the older group might be explained by a higher prevalence of antithrombotic drugs, whereas the more frequent cases of lead dislodgments may have been caused by atrial and ventricular dilatation due to the higher incidences of atrial fibrillation and heart failure in contrast to younger patients [35].

### 4.3. Complications and Mortality during Follow-Up

The rates for device-associated rehospitalization and revision surgery during follow-up, with 22.6% (vs. 13.9%, *p* < 0.001) and 13.7% (vs. 8.1%, *p* = 0.002), were significantly higher in younger patients compared to older patients receiving ICD/CRT-D. Meta-analyses and registry data on ICD therapy show a broad range of device-associated complication rates, between 9.1% within 16 months and 9.5% in 6 months [36,37]. Christensen et al. focused on young ARVC patients receiving ICD and reported an overall complication rate of 7.9% and a rate of 5.5% for revision surgery within the first year after implantation [38]. Olde Nordkamp et al. [10] conducted a meta-analysis on young ICD recipients with inherited arrhythmia syndromes and reported an even lower device-related complication rate of 22% over 5 years (4.4%/year), with lead dysfunction as the most common cause; this is consistent with findings from lead extraction studies in the young [11]. The higher incidence of lead fractures in younger patients is often attributed to a more active physical lifestyle, unlimited by heart failure symptoms; this is underlined in our study by the high proportion (51.7%) of patients rejoining the workforce (Table 4). Rosenkranz et al. were able to demonstrate from Danish national registry data that comparable proportions of patients in the age group of 30–45 years rejoin the workforce 1 year after ICD implantation (52% for primary prevention and 66% for secondary prevention) [39]. Our subgroup analysis of younger CHF patients with mostly primary preventive ICDs revealed a markedly lower rate for revision surgeries, with 9.5% during FU, which might also support this notion. Another reason for the high revision rate in our study might have been the temporary popularity of the now recalled small-diameter ICD leads, such as Sprint Fidelis (Medtronic, Minneapolis, MN, USA) or Riata (Abbott/St. Jude Medical, St. Paul, MN, USA) leads, which promised easier insertion and less lead-associated complications, but proved to be the opposite with high incidences of premature lead failure, especially in young patients [40]. The introduction of the S-ICD was supposed to mitigated fears of lead dysfunction or infection and was able to provide young patients without pacing indication, with a potentially safer alternative to conventional transvenous ICD (tv-ICD) [14]. In 2020, the PRAETORIAN trial was able to demonstrate the non-inferiority of the S-ICD compared to tv-ICD, regarding device-associated complications and inappropriate shocks (IASs) [41].

During our FU, 13.5% of young patients suffered from an ICD shock within 1 year. Unfortunately, our data do not discriminate between appropriate and inappropriate shock therapies, which grossly limits the scope of interpretation. The meta-analysis from Olde Nordkamp et al. reported an annual rate for appropriate therapy of 6.1% and inappropriate therapy of 4.7% in young ICD patients [10]. In 2012, the MADIT-RIT trial could demonstrate that less aggressive programming with long detection cycles and high-rate cut-offs was able to decrease IAS rate dramatically from 29% to 6% at 2.5 years FU while maintaining excellent patient safety and even reducing cumulative mortality [13]. It is fair to assume that a reasonable number of IASs could have been prevented if modern device programming had been applied.

The 1-year mortality of younger patients was 2.9% vs. 7.3% in older patients, which seems to be mostly attributable to the higher incidences of comorbidities and thus higher incidence of MACCE (3.4 vs. 8.3%) during FU in the older patient group. The meta-analysis by Olde Nordkamp et al. of young ICD patients showed slightly lower mortality rates with 4.3% after a mean FU of 51 ± 38 months, but only included patients with HCM and inherited arrhythmia syndromes, thus excluding young DCM and ICM patients with potentially worse prognosis [10]. The mortality rate of 5.5% within 6 months for unfiltered ICD recipients from a national Danish registry seems comparable to our older patient group [37]. In our subgroup analysis of CHF patients, the mortality benefit for younger patients is entirely lost (6.2 vs. 8.3%; HR: 0.74, 95% CI: 0.31–1.81), which underlines the high mortality of heart failure, regardless of comorbidities and age [42].

## 5. Conclusions

Young recipients of defibrillator therapy in Germany more often present with HCM or primary electrical diseases compared to older patients. Defibrillator implantation in younger patients is more frequently provided for secondary prevention. There were no differences in procedural or intra-hospital complications during index hospitalization, but there was an increased incidence of device-related rehospitalization during follow-up, despite better survival in younger patients. More research on the age-specific risk factors and needs during post-implantation care of young defibrillator recipients is warranted in order to improve patient safety and satisfaction.

## 6. Limitations

This study has several limitations: the registry-based study design and data procurement made this study vulnerable to reporting bias, as well as selection and detection bias. Device indication, implantation, and post-procedural care were at the discretion of the implanting centers and thus not standardized. Therefore, the influence of individual physician decisions and variations in care cannot be eliminated. The follow-up was centralized, limited to 1 year, and only performed via telephone call, with a standardized but limited set of questions. Specific data on quality of life (e.g., Kansas City Cardiomyopathy Questionnaire—KCCQ) or detailed data on appropriate and inappropriate shocks are missing. When patient inclusion began in 2007, crucial heart failure therapeutics and treatment concepts, as well as novel devices, which have become standard of care in the most recent years, had not been introduced into the therapeutic armamentarium for heart failure and SCD prevention. Thus, the results of this study cannot be fully transferred into contemporary patient populations and must be carefully interpreted within the temporal context the data were obtained. Nevertheless, the German DEVICE registry give valuable insight into a large, prospective real-world patient population and adds important data on young patients undergoing ICD/CRT-D implantation.

## Figures and Tables

**Table 1:** Patient characteristics.

	Patient Age < 45 (n = 339)	Patient Age ≥ 45 (n = 4974)	*p*-Value
Age, years, mean ± SD	35.0 ± 8.2	67.5 ± 9.7	<0.001
Female sex, % (n)	28.9% (98/339)	18.4% (916/4974)	<0.001
BMI, kg/m^2^, median [IQR]	25.0 [21.9–28.5]	27.1 [24.6–30.2]	0.001
LVEF, %, median [IQR]	40 [25–60]	30 [25–35)	<0.001
Underlying cardiac disease, % (n)	70.8% (240/339)	93.8% (4668/4974)	<0.001
History of MI, % (n)	8.3% (28/339)	36.6% (1821/4974)	<0.001
Prior myocardial revascularization (PCI or CABG), % (n)	6.7% (7/105)	46.5% (519/1117)	<0.001
Congestive heart failure, % (n)	27.1% (92/339)	51.6% (2569/4974)	<0.001
**ECG at admission**			
- Sinus rhythm % (n)	93.2% (316/339)	76.9% (3822/4968)	<0.001
- Atrial fibrillation, % (n)	3.5% (12/339)	19.6% (975/4968)	<0.001
- AV conduction disturbances, % (n)	7.7% (26/339)	18.0% (894/4961)	<0.001
- LBBB, % (n)	11.8% (40/339)	33.5% (1661/4962)	<0.001
- Median QRS duration, ms [IQR]	100 [90–120]	120 [100–150]	<0.001
- QRS ≥ 150 ms, % (n)	11.0% (37/337)	29.1% (1442/4951)	<0.001
**Comorbidities**			
- History of CVA, % (n)	2.1% (7/339)	4.2% (207/4973)	0.057
- PAD, % (n)	0.6% (2/339)	3.3% (163/4973)	0.006
- Diabetes mellitus, % (n)	3.5% (12/339)	28.7% (1428/4973)	<0.001
- Arterial hypertension, % (n)	15.6% (53/339)	55.1% (2740/4973)	<0.001
- COPD, % (n)	0.6% (2/339)	3.9% (196/4973)	0.002
- Chronic kidney disease, % (n)	5.0% (17/339)	18.1% (901/4973)	<0.001

BMI: body mass index; CABG: coronary artery bypass graft, ECG: electrocardiogram; LBBB: left-bundle branch block; LVEF: left-ventricular ejection fraction; MI: myocardial infarction; PAD: peripheral artery disease; PCI: percutaneous coronary intervention; SD: standard deviation—all *p*-value < 0.05 was considered statistically significant.

**Table 2:** Device indication.

	Patient Age < 45 (n = 339)	Patient Age ≥ 45 (n = 4974)	*p*-Value
Primary prevention, % (n)	48.4% (164/339)	60.1% (2991/4974)	<0.001
Secondary prevention, % (n)	51.6% (175/339)	39.9% (1983/4974)	<0.001
Causative arrhythmia *			
- Ventricular fibrillation (VF), % (n)	60.6% (106/175)	37.9% (752/1983)	<0.001
- Ventricular tachycardia (VT), % (n)	25.7% (45/175)	46.8% (928/1983)	<0.001
- Syncope with inducible VF/VT, % (n)	11.4% (20/175)	13.2% (262/1983)	0.50
- Other arrhythmia	2.3% (4/175)	2.1% (41/1983)	0.85
Leading cause for initial admission *			
- Cardiopulmonary resuscitation, % (n)	66.7% (106/159)	45.4% (765/1685)	<0.001
- Syncope, % (n)	20.1% (32/159)	24.7% (416/1685)	0.20
- Presyncope, % (n)	5.0% (8/159)	15.7% (265/1685)	<0.001
- Other, % (n)	8.2% (13/159)	14.2% (239/1685)	0.035
Family history of SCD, % (n)	15.2% (16/105)	3.9% (43/1112)	<0.001

* for patients with secondary prevention; SCD: sudden cardiac death. A *p*-value < 0.05 was considered statistically significant.

**Table 3:** Procedural data and index hospitalization.

	Patient Age < 45 (n = 339)	Patient Age ≥ 45 (n = 4974)	*p*-Value
Urgent procedure, % (n)	14.4% (15/104)	8.7% (97/1116)	0.053
Elective procedure, % (n)	85.6% (89/104)	91.3% (1019/1116)	0.053
Procedural duration, min, median [IQR]	46 [37–81]	62 [42–105]	<0.001
**Device type**			
- Single chamber (VVI) ICD, % (n)	75.2% (255/339)	49.1% (2441/4974)	<0.001
- Dual chamber (DDD) ICD, % (n)	15.6% (53/339)	20.8% (1036/4974)	0.022
- CRT-D, % (n)	9.1% (31/339)	30.1% (1497/4974)	<0.001
**Device Manufacturer**			
- Biotronik	18.3% (62/339)	21.7% (1080/4969)	0.14
- ELA Medical	0.6% (2/339)	2.1 % (102/4969)	0.060
- Guidant/Boston Scientific	17.4% (59/339)	15.1% (748/4939)	0.24
- Medtronic	26.5% (90/339)	31.7% (1576/4969)	0.047
- St. Jude Medical	25.7% (87/339)	27.6% (1370/4969)	0.45
- Others	11.5% (39/339)	1.9% (93/4969)	<0.001
Defibrillation threshold testing, % (n)	85.6% (274/320)	73.5% (3437/4675)	<0.001
- Successful, % (n)	99.6% (273/274)	99.4% (3416/3437)	0.61
- Unsuccessful, % (n)	0.4% (1/274)	0.6% (21/3437)	0.61
Complications with interventions, % (n)	1.5% (5/339)	1.9% (96/4946)	0.68 *
- Pericardial tamponade/effusion, % (n)	0.3% (1/338)	0.1% (6/4946)	0.37 *
- Hemothorax, % (n)	0.0% (0/338)	0.1% (5/4946)	1.00 *
- Pneumothorax, % (n)	0.9% (3/339)	0.4% (22/4946)	0.21 *
- Pocket hematoma, % (n)	0.3% (1/338)	1.3% (63/4946)	0.19 *
Revision procedure during index hospitalization, % (n)	1.8% (4/228)	2.2% (82/3715)	0.82 *
Median duration of hospital stay, days [IQR]	3 [2–5]	3 [2–5]	0.28
Intrahospital mortality, % (n)	0.0% (0/339)	0.3% (16/4970)	0.62 *
Antiarrhythmic drugs at discharge, % (n)	10.6% (36/339)	19.4% (962/4955)	<0.001

CRT-D/-P: cardiac resynchronization therapy defibrillator/-pacemaker; ICD: implantable cardioverter–defibrillator. A *p*-value < 0.05 was considered statistically significant. * Fisher’s exact test.

**Table 4:** Follow-up data.

	Patient Age < 45 (n = 339)	Patient Age ≥ 45 (n = 4974)	*p*-Value
Follow-up completed, % (n)	93.5% (319/339)	97.0 % (4823/4974)	<0.001
Duration of FU, months, median [IQR] *	17.9 [13.4; 22.9)	16.9 (13.1; 23.1)	0.13
1-year mortality, % **	2.9	7.3	0.004
MACCE (Death, MI, Stroke)	3.4 % (11/319)	8.3 % (410/4937)	0.002
Survivors, n	n = 328	n = 4280	
Rehospitalization, % (n)	43.4% (108/249)	41.4% (1479/3575)	0.54
Device-associated rehospitalization, % (n)	22.6% (56/248)	13.9% (496/3561)	<0.001
Non-cardiovascular rehospitalization, % (n)	10.1% (25/248)	16.0% (570/3561)	0.013
Median cumulative duration of all rehospitalizations, days [IQR]	7 [4–14]	10 [5–20]	0.023
Defibrillator shock, % (n)	19.2% (51/266)	17.0% (626/3676)	0.37
Revision surgery, % (n)	13.7% (35/255)	8.1% (283/3500)	0.002
Antiarrhythmic drugs at follow-up, % (n)	13.5% (33/245)	23.1% (804/3475)	<0.001
**Employment status**			
- Employed	51.7% (77/149)	10.6% (204/1916)	<0.001
- Retired	16.1% (24/149)	73.2% (1403/1916)	<0.001
- Other/Unknown	34.2% (48/149)	16.2% (308/1916)	<0.001
**Patient satisfaction/assessment ^+^**			
- Therapy successful, % (n)	92.1% (117/127)	91.1% (1392/1528)	0.70
- Therapy partially successful, % (n)	7.1% (9/127)	7.0% (107/1528)	
- Therapy unsuccessful, % (n)	0.8 % (1/127)	1.9% (29/1528)	0.37
- ICD provides SCD protection, % (n)	95.9% (71/74)	93.8% (710/746)	0.84
- Patient concerns about ICD shocks, % (n)	48.6% (35/72)	20.5% (152/742)	<0.001

* Time from implantation until first FU contact; ** Kaplan–Meier estimates, log-rank test, Hazard ratio: 0.39; 95% confidence interval: 0.20–0.75. FU: follow-up; ^+^ Patient evaluation of therapy during FU interview; ICD: implantable cardioverter–defibrillator; MI: myocardial infarction; SCD: sudden cardiac death.

## Data Availability

The data pertaining this study will be made available by the corresponding author upon reasonable request.

## Supplementary Materials

The following supporting information can be downloaded at: https://www.mdpi.com/article/10.3390/jcm13133858/s1. Figure S1: Bar diagrams displaying the proportion of patients with antiarrhythmic drugs at dis-charge and after follow-up (FU). (A) displaying the distribution of different classes of antiarrhythmic drugs across age groups at discharge and FU. (B) displaying the proportion of different class III drugs across age groups at discharge and FU.
