# Supplementary material for: Procedural Outcome and 1-Year Follow-Up of Young Patients Undergoing Implantable Cardioverter–Defibrillator Implantation—Insights from the German DEVICE I+II Registry
Source: J Clin Med. 2024 Jun 30;13(13):3858. doi: 10.3390/jcm13133858 (PMC11242717; doi:10.3390/jcm13133858)
Supplement: Supplementary file 1 [file jcm-13-03858-s001.zip › jcm-2988419-supplementary.pdf]

Supplementary Materials

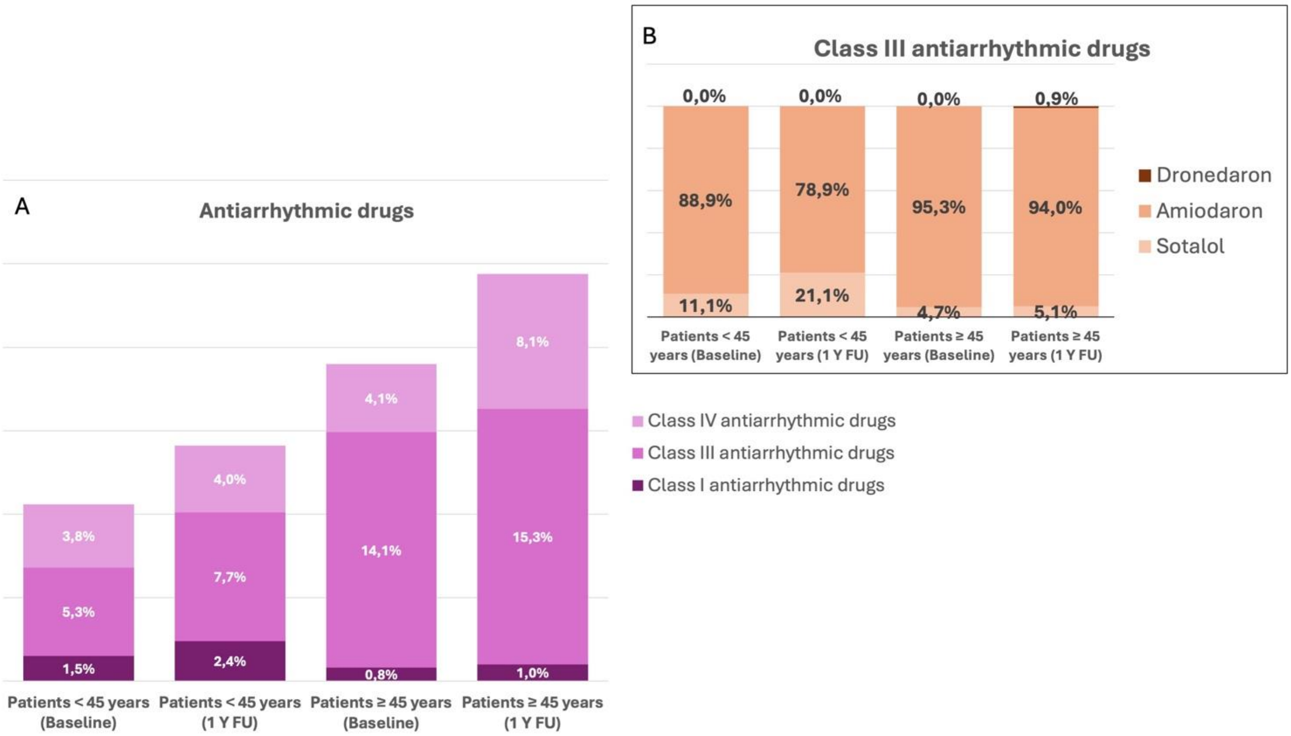

Figure S1. – Bar diagrams displaying the proportion of patients with antiarrhythmic drugs at discharge and after follow-up (FU). A) displaying the distribution of different classes of antiarrhythmic drugs across age groups at discharge and FU. B) displaying the proportion of different class III drugs across age groups at discharge and FU.
